# Measured sodium excretion is associated with cardiovascular outcomes in non-dialysis CKD patients: results from the KNOW-CKD study

**DOI:** 10.3389/fneph.2023.1236177

**Published:** 2023-08-25

**Authors:** Seong Cheol Kang, Minjung Kang, Hyunjin Ryu, Seonmi Kim, Ji Hye Kim, Eunjeong Kang, Yujin Jeong, Jayoun Kim, Yong-Soo Kim, Soo Wan Kim, Yeong Hoon Kim, Kook-Hwan Oh

**Affiliations:** ^1^ Department of Medical Sciences, Seoul National University College of Medicine, Seoul, Republic of Korea; ^2^ Department of Internal Medicine, Seoul National University Hospital, Seoul, Republic of Korea; ^3^ Department of Internal Medicine, Chungbuk National University Hospital, Cheongju, Republic of Korea; ^4^ Transplantation Center, Seoul National University Hospital, Seoul, Republic of Korea; ^5^ Department of Biostatistics, Korea University College of Medicine, Seoul, Republic of Korea; ^6^ Medical Research Collaborating Center, Seoul National University Hospital, Seoul, Republic of Korea; ^7^ Department of Internal Medicine, College of Medicine, The Catholic University of Korea, Seoul, Republic of Korea; ^8^ Department of Internal Medicine, Chonnam National University Medical School, Gwangju, Republic of Korea; ^9^ Department of Internal Medicine, Inje University Busan Paik Hospital, Busan, Republic of Korea; ^10^ Kidney Research Institute, Seoul National University Medical Research Center, Seoul, Republic of Korea; ^11^ Department of Internal Medicine, Seoul National University College of Medicine, Seoul, Republic of Korea

**Keywords:** cardiovascular outcome, major adverse cardiac event, all-cause mortality, chronic kidney disease, dietary salt intake

## Abstract

**Background:**

There are insufficient studies on the effect of dietary salt intake on cardiovascular (CV) outcomes in chronic kidney disease (CKD) patients, and there is no consensus on the sodium (Na) intake level that increases the risk of CV disease in CKD patients. Therefore, we investigated the association between dietary salt intake and CV outcomes in CKD patients.

**Methods:**

In the Korean cohort study for Outcome in patients with CKD (KNOW-CKD), 1,937 patients were eligible for the study, and their dietary Na intake was estimated using measured 24h urinary Na excretion. The primary outcome was a composite of CV events and/or all-cause death. The secondary outcome was a major adverse cardiac event (MACE).

**Results:**

Among 1,937 subjects, there were 205 (10.5%) events for the composite outcome and 110 (5.6%) events for MACE. Compared to the reference group (urinary Na excretion< 2.0g/day), the group with the highest measured 24h urinary Na excretion (urinary Na excretion ≥ 8.0g/day) was associated with increased risk of both the composite outcome (hazard ratio 3.29 [95% confidence interval 1.00-10.81]; P = 0.049) and MACE (hazard ratio 6.28 [95% confidence interval 1.45-27.20]; P = 0.013) in a cause-specific hazard model. Subgroup analysis also showed a pronounced association between dietary salt intake and the composite outcome in subgroups of patients with abdominal obesity, female, lower estimated glomerular filtration rate (< 60 ml/min per 1.73m^2^), no overt proteinuria, or a lower urinary potassium-to-creatinine ratio (< 46 mmol/g).

**Conclusion:**

A high-salt diet is associated with CV outcomes in non-dialysis CKD patients.

## Introduction

Chronic kidney disease (CKD) is a risk factor for cardiovascular (CV) disease and all cause-mortality (1, 2). CV disease is the leading cause of death among CKD patients (3), and CV mortality accounts for about 50% of deaths in patients with advanced CKD, compared to 26% in the general population (4). Therefore, it is important to reduce the risk of CV disease in CKD patients. Blood pressure control with angiotensin-converting enzyme inhibitors and angiotensin receptor blockers (5) and lifestyle changes such as a low-salt diet (6), weight loss (7), physical activity (8), and smoking cessation (9) are known modifiable factors that affect the risk of CV diseases. As a part of lifestyle modifications for CKD patients, controlling dietary salt intake is a crucial modifiable factor for reducing CV events (10).

According to the World Health Organization (WHO) guideline, reducing dietary sodium (Na) intake can decrease blood pressure and the risk of CV disease, and the WHO recommends a dietary Na intake less than 2.0g/day, which is equivalent to 5.0g/day of salt (11). Also, many studies have demonstrated the associations between dietary salt intake and CV events and mortality in the general population (12–14), and they showed a positive association between Na intake and CV diseases with a high-salt diet. However, the guideline and studies are based on data from the general population (11–14), and there are insufficient studies that focused on CKD patients to assess the association between salt intake and CV disease or mortality. In addition, there is no consensus on the level of Na intake that increases the risk of CV disease in CKD patients. In the Chronic Renal Insufficiency Cohort (CRIC) Study, the threshold of Na intake above which the risk of CV events increased in CKD patients was 4.5g/day (12), and those from other studies on CKD patients were 4.0g/day (15) and 4.8g/day (15, 16). Dietary salt intake varies widely according to culture and race (17), but no studies have identified the association between salt intake and CV risk among Korean CKD patients.

This study aimed to assess the effect of dietary salt intake on CV disease and the cut-off value of salt intake that affected CV disease in CKD patients by analyzing data from the KoreaN cohort study for Outcome in patients with CKD (KNOW-CKD).

## Materials and methods

### Study participants

The KNOW-CKD study is an ongoing, nationwide, multicenter, prospective long-term cohort study of CKD patients used to identify clinical outcomes of CKD patients, aggravating factors of renal function, and factors associated with complications such as CV diseases (18). Among 2,238 CKD subjects enrolled in the KNOW-CKD cohort from 2011 to 2016, patients who previously underwent organ transplant or dialysis, patients with heart failure (New York Heart Association class III or IV), patients with cancer and liver cirrhosis, and pregnant patients were excluded. Patients without a urine sample (n=56), with an incomplete 24h urine specimen in which the collected urine was less than 500ml (n=5), without baseline 24h urine Na excretion (n=226), or with urinary Na excretion at extreme values (≥ 1,000mEq/day or< 20mEq/day) (n=11) were excluded. Finally, subjects with follow-up loss (n=3) were excluded, leaving 1,937 subjects in the study (**Supplementary Figure 1**). Subjects were divided into five groups according to measured 24h urinary Na excretion. The study was designed and conducted in accordance with the Principles of the Declaration of Helsinki. The study was approved by the Institutional Review Boards of Seoul National University Hospital (1104-089-359), Seoul National University Bundang Hospital (B-1106/129-008), Yonsei University Severance Hospital (4-2011-0163), Kangbuk Samsung Medical Center (2011-01-076), Seoul St. Mary’s Hospital (KC11OIMI0441), Gil Hospital (GIRBA2553), Eulji General Hospital (201105-01), Chonnam National University Hospital (CNUH-2011-092), and Busan Paik Hospital (11-091).

### Data collection and measurements

In order to identify baseline characteristics at study entry, demographic information and medical history were collected using questionnaires and medical records. History of CV disease was defined as any history of previous CV disease such as cerebrovascular disease, coronary artery disease, peripheral vascular disease, congestive heart failure, and arrhythmia. Abdominal obesity was defined as waist-to-hip ratio ≥ 0.95 for men and ≥ 0.8 for women (19). Uncontrolled hypertension was defined as systolic blood pressure (SBP) ≥ 140 mmHg or diastolic blood pressure (DBP) ≥ 90 mmHg. Also, serum hemoglobin, albumin, total cholesterol, low-density lipoprotein (LDL) cholesterol, high-density lipoprotein (HDL) cholesterol, triglycerides, and fasting blood sugar were measured as baseline laboratory findings. Baseline urine protein was measured through collected 24h urine samples, and 24h urinary Na excretion was measured to estimate dietary Na intake. Measured 24h urinary Na excretion was divided as follows:< 2.0g/day, 2.0≤ and<4.0g/day, 4.0≤ and<6.0g/day, 6.0≤ and<8.0g/day, and ≥ 8.0g/day. For measurement of spot urinary Na, protein, potassium, and creatinine, second-voided urine samples were used. The estimated GFR (eGFR) was calculated using the Chronic Kidney Disease Epidemiology Collaboration (CKD-EPI) equation (20).

### Outcomes

The primary outcome was the composite of all-cause death or occurrence of CV events. CV events were defined as myocardial infarction, unstable angina, coronary revascularization, heart failure, stroke, and other CV events. The secondary outcome was major adverse cardiac events (MACE), defined as the composite of fatal CV death, acute myocardial infarction, hospitalization because of heart failure, unstable angina, stroke, and symptomatic arrhythmia. Survival time was calculated from study entry until diagnosis of CV events or all-cause death.

### Statistical analyses

All analyses were performed using SPSS (version 26.0; IBM Corporation, Armonk, NY, USA) and R software (version 4.1.1; www.r-projectorg; R Foundation for Statistical Computing, Vienna, Austria). In baseline characteristics, continuous variables were expressed as medians (interquartile ranges), and categorical variables were expressed as numbers (%). To test normality, the Shapiro-Wilk test was used. To calculate P for trend, we used the Jonckheere-Terpstra test for continuous variables and the linear-by-linear test for categorical variables. Cause-specific hazard models were used to explore the associations between urinary Na excretion and risks for the primary and secondary outcomes. Development of end-stage kidney disease (ESKD) before the primary outcome was the competing event. ESKD was defined as the initiation of renal replacement therapy. Multivariable models were used. In model 1, demographic factors (age, sex, body mass index [BMI], and urinary creatinine excretion) were adjusted. In model 2, baseline estimated GFR and spot urinary potassium-to-creatinine ratio were adjusted in addition to the factors of model 1. Finally, in model 3, we additionally adjusted for etiology of CKD, presence of diabetes mellitus (DM), use of medications (diuretics and renin-angiotensin system blockers), systolic blood pressure (SBP), smoking, and random urine protein-to-creatinine ratio. We used a cubic spline curve to identify the associations between urinary Na excretion and the primary and secondary outcomes, and model 3 was applied to the curve. A Ward-test was conducted to confirm the non-linearity of this association between urinary Na excretion and the primary outcome. We performed subgroup analyses in accordance with age, sex, DM, uncontrolled hypertension, abdominal obesity, eGFR, proteinuria, and random urinary potassium-to-creatinine ratio. For sensitivity analysis, we obtained estimated 24h urinary Na excretion using the Pan American Health Organization (PAHO) formula with random urine Na and creatinine (21). Excluding those with missing values, 2,096 patients were analyzed for sensitivity analysis. *P* values< 0.05 were considered significant.

## Results

### Baseline characteristics

**Table 1** shows the baseline characteristics of participants according to 24h urinary Na excretion. The median age of the participants was 55 years, and male subjects held a majority in most groups. The median 24h urinary Na excretion was 151.0 mEq/day. Subjects with higher urinary Na excretion were more likely to be current or former smokers (P for trend< 0.001). Also, higher urinary Na excretion had relevance with higher body mass index (P for trend< 0.001) and diastolic BP (P for trend = 0.001). Regarding SBP, in the section where urinary Na excretion<8g/day, SBP tends to increase as urinary Na excretion increases, and the highest values were observed in urinary Na excretion between 6 and 8 g/day. SBP has a relatively low value in the section where urinary Na excretion ≥8g/day. As for laboratory values, eGFR (P for trend< 0.001) and hemoglobin (P for trend< 0.001) increased with urinary Na excretion. Lastly, higher urinary Na excretion was associated with higher urinary protein (P for trend< 0.001), urinary creatinine (P for trend< 0.001), and urinary potassium (P for trend< 0.001) (**Table 1**).

**Table 1:** Baseline participant characteristics at study entry.

Variable	Measured 24-hour urinary sodium excretion (g/day)						
	Total (n = 1937)	<2 (n = 261)	2≤<4 (n = 978)	4≤<6 (n = 536)	6≤<8 (n = 137)	≥8 (n = 25)	*P for trend*
age, years	55[45;63.5]	55.0 [46.0;63.0]	55.0 [46.0;64.0]	55.0 [46.0;64.0]	53.0 [44.0;60.0]	48.0 [44.0;55.0]	0.055
Male, n(%)	1367(61.1)	126 (48.3)	537 (54.9)	380 (70.9)	114 (83.2)	20 (80.0)	<0.001
Etiology of CKD, n(%)							0.781
Glomerulonephritis	810(36.2)	80 (30.7)	334 (34.2)	193 (36.0)	53 (38.7)	9 (36.0)	
Diabetic nephropathy	518(23.1)	68 (26.1)	231 (23.6)	124 (23.1)	30 (21.9)	5 (20.0)	
Hypertensive, non-glomerular nephropathy	409(18.3)	50 (19.2)	169 (17.3)	109 (20.3)	22 (16.1)	4 (16.0)	
Polycystic kidney disease	364(16.3)	48 (18.4)	194 (19.8)	71 (13.2)	21 (15.3)	2 (8.0)	
Other diseases	137(6.1)	15 (5.7)	50 (5.1)	39 (7.3)	11 (8.0)	5 (20.0)	
Smoking, n(%)							<0.001
never	1201(53.7)	168 (64.4)	558 (57.1)	258 (48.2)	40 (29.2)	5 (20.0)	
former	677(30.3)	17 (6.5)	143 (14.6)	107 (20.0)	28 (20.4)	7 (28.0)	
current	350(15.6)	76 (29.1)	277 (28.3)	170 (31.8)	69 (50.4)	13 (52.0)	
History of CVD, n(%)	40 (15.3%)	40 (15.3)	165 (16.9)	91 (17.0)	18 (13.1)	1 (4.0)	0.401
Diabetes mellitus, n(%)	754(33.7)	90 (34.5)	323 (33.0)	197 (36.8)	46 (33.6)	9 (36.0)	
Body mass index, kg/m²	24.4[23.3;26.5]	23.2[21.0;25.6]	24.2[22.0;26.1]	25.0[23.2;27.3]	25.5[23.9;28.1]	27.1[25.3;28.7]	<0.001
Systolic BP, mmHg	127[118;137]	124.0 [115.0;135.0]	126.0 [117.0;136.0]	128.0 [119.0;138.0]	130.0 [120.0;145.0]	126.0 [117.0;133.5]	0.001
Diastolic BP, mmHg	77[69;84]	74.0 [67.0;81.0]	77.0 [69.0;84.0]	77.0 [70.0;84.0]	79.0 [73.0;88.0]	79.5 [67.0;86.5]	0.001
Use of medication							
Diuretics, n(%)	612(31.6)	82 (31.4)	295 (30.2)	181 (33.8)	47 (34.3)	7 (28.0)	0.327
RAS blocker, n(%)	1666(86.0)	215 (82.4)	826 (84.5)	479 (89.4)	124 (90.5)	22 (88.0)	0.001
Laboratory findings							
eGFR, ml/min/1.73 m^2^	46.7[28.6;74.0]	47.8 [31.1;68.1]	57.6 [37.8;75.7]	59.6 [38.7;77.7]	63.5 [47.1;83.5]	68.4 [42.6;92.1]	<0.001
Hemoglobin, g/dL	12.8[11.4;14.4)	12.8[11.2;14.2]	12.7[11.4;14.4]	12.9[11.4;14.6]	12.9[11.2;14.2]	13.8[11.4;15.2]	0.174
Albumin, g/dL	4.2[4.0;4.5]	4.2 [4.0; 4.5]	4.2 [4.0; 4.5]	4.3 [4.0; 4.5]	4.3 [4.0; 4.5]	4.3 [3.9; 4.5]	0.546
Total cholesterol, mg/dL	171.0[146.0;198.0]	174.5[147.0;200.0]	169.0[146.0;196.0]	172.0[147.0;198.0]	171.0[144.5;198.0]	173.5[152.8;201.8]	0.763
LDL cholesterol, mg/dL	93.0[13.0;116.0]	92.5[73.3;115.0]	91.0[72.0;114.0]	95.0[75.0;116.0]	94.0[72.0;117.0]	102.0[81.5;121.5]	0.342
HDL cholesterol, mg/dL	47.0[38.4;58.0]	48.0[39.0;59.3]	47.0[39.0;58.0]	46.0[38.0;57.7]	45.0[36.0;58.0]	42.0[36.5;51.0]	0.086
Triglyceride, mg/dL	132.0[91.0;194.0]	129.0[91.0;184.0]	133.5[90.1;193.0]	132.0[91.0;201.0]	135.0[94.0;228.0]	164.0[117.0;217.0]	0.066
Fasting blood sugar, mg/dL	100.0[92.0;115.0]	98.0[90.0;112.3]	100.0[92.0;115.0]	100.0[93.0;113.0]	101.0[93.0;118.0]	96.0[87.3;125.3]	0.123
24h measured urinary Na excretion, mEq/day	151.0[109.5;500]	67.3 [55.0;78.0]	132.9 [112.0;153.0]	208.3 [189.0;228.9]	283.9 [271.0;307.2]	372.5 [355.8;396.0]	<0.001
urine protein, mg/day	540[186.9;1574.5]	429.0 [138.0;1119.0]	500.0 [156.2;1335.7]	653.5 [199.8;1932.0]	986.9 [209.3;3536.0]	1610.0 [322.6;5126.5]	<0.001
Urinary creatinine, mg/day	1124.0[888.4;1455.0]	852.0[622.5;1101.0]	1020.0 [842.0;1313.8]	1320.0 [1085.0;1600.0]	1570.0 [1316.0;1834.0]	1572.0 [1334.0;2006.2]	<0.001
Urinary potassium, mg/day	50.1[37.0;66.0]	33.7 [24.0;45.6]	47.0 [36.0;59.0]	59.9 [48.0;73.0]	77.0 [63.0;87.2]	92.4 [74.2;132.9]	<0.001

CKD, chronic kidney disease; CVD, cardiovascular disease; eGFR, estimated glomerular filtration rate; LDL, low-density lipoprotein; HDL, high density lipoprotein. Continuous variables were expressed as medians (interquartile ranges), and categorical variables were expressed as numbers (%). To calculate P for trend, we used the Jonckheere-Terpstra test for continuous variables and the linear-by-linear test for categorical variables.

### Cardiovascular outcomes and MACE

**Figure 1:**
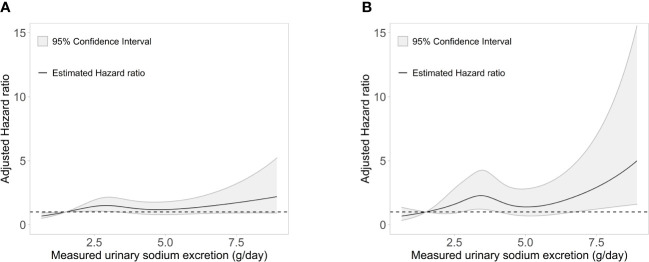
Association of measured 24-h urinary sodium excretion with the HR of **(A)** the composite outcome of cardiovascular event and all-cause death and **(B)** major adverse cardiac events. HR, hazard ratio.

During a median (interquartile range) follow-up of 7.0 years (5.0-8.7 years), a total of 205 (10.5%) events for the composite outcome of CV events and all-cause death occurred (**Table 2**). With the reference being the lowest Na excretion group (24h urinary Na excretion<2.0g/day), the hazard ratios (HRs) for the composite outcome for the highest excretion group (24h urinary Na excretion ≥8.0g/day) were 3.12 (1.04-9.35), 4.97 (1.56-15.86), and 3.29 (1.00-10.81) in models 1, 2, and 3, respectively. In other groups, urinary Na excretion was not significantly related to the composite outcome. The spline curve showed a non-linear association between urinary Na excretion and the composite outcome of CV event and all-cause death, and the HR was particularly higher when 24h urinary Na excretion was 8g/day or above (**Figure 1A**).

**Table 2:** Association of urinary sodium excretion with the composite outcome of cardiovascular event and all-cause death.

24h urine sodium excretion (g/day)	Number of participants	Number of events (%)	Model 1		Model 2		Model 3	
			HR (95% CI)	*P*	HR (95% CI)	*P*	HR (95% CI)	*P*
<2	261	20(7.6)	1 (Reference)		1 (Reference)		1 (Reference)	
2≤<4	978	112 (11.4)	1.52 (0.93-2.47)	0.090	1.61 (0.97-2.67)	0.061	1.47 (0.89-2.44)	0.131
4≤<6	536	57(10.6)	1.49 (0.86-2.56)	0.147	1.55 (0.88-2.72)	0.127	1.27 (0.71-2.27)	0.405
6≤<8	137	12(8.7)	1.50 (0.70-3.25)	0.293	1.61 (0.72-3.62)	0.244	1.19 (0.52-2.73)	0.664
≥8	25	4 (16.0)	3.12 (1.04-9.35)	0.041	4.97 (1.56-15.86)	0.006	3.29 (1.00-10.81)	0.049
Total	1937	205(10.5)						

HR, Hazard ratio; CI, confidence interval.

Model 1: Age, sex, body mass index, and urinary creatinine excretion.

Model 2: Model 1 plus baseline estimated glomerular filtration rate, and spot urinary potassium-to-creatinine ratio.

Model 3: Model2 plus etiology of CKD, presence of DM, use of diuretics, use of RAS blockers, systolic blood pressure, smoking, and random urine protein-to-creatinine ratio.

For MACE, from the groups with the lowest to the highest urinary Na excretion, 9 (3.4%), 64 (6.5%), 26 (4.8%), 8 (5.8%), and 3 (12.0%) events occurred, respectively, with a total of 110 (5.6%) events (**Table 3**). With the reference being the lowest group of Na excretion, the HR of the highest Na excretion group in model 3 was 6.28 (1.45-27.20), with a p value of 0.013. The rest of the groups did not show statistical significance in any models. Also, the spline curve demonstrated a positive association between urinary Na excretion and MACE (**Figure 1B**).

**Table 3:** Association of urinary sodium excretion with major adverse cardiac events (MACE).

24h urine sodium excretion (g/day)	Number of participants	Number of events (%)	Model 1		Model 2		Model 3	
			HR (95% CI)	*P*	HR (95% CI)	*P*	HR (95% CI)	*P*
<2	261	9(3.4)	1 (Reference)		1 (Reference)		1 (Reference)	
2≤<4	978	64(6.5)	1.87 (0.92-3.81)	0.081	1.88 (0.91-3.86)	0.084	1.71 (0.82-3.53)	0.145
4≤<6	536	26(4.8)	1.50 (0.68-3.34)	0.311	1.44 (0.63-3.27)	0.380	1.21 (0.52-2.78)	0.655
6≤<8	137	8(5.8)	2.31 (0.83-6.41)	0.108	2.25 (0.76-6.60)	0.139	1.64 (0.54-4.92)	0.377
≥8	25	3 (12.0)	6.42 (1.66-24.72)	0.006	9.32 (2.21-39.32)	0.002	6.28 (1.45-27.20)	0.013
Total	1937	110(5.6)						

MACE was defined as the composite of fatal cardiovascular death, acute myocardial infarction, hospitalization because of heart failure and unstable angina, stroke, and symptomatic arrhythmia.

HR, Hazard ratio; CI, confidence interval.

Model 1: Age, sex, body mass index, and urinary creatinine excretion.

Model 2: Model 1 plus baseline estimated glomerular filtration rate, and spot urinary potassium-to-creatinine ratio.

Model 3: Model2 plus etiology of CKD, presence of DM, use of diuretics, use of RAS blockers, systolic blood pressure, smoking, and random urine protein-to-creatinine ratio.

### Subgroup analyses

**Figure 2:**
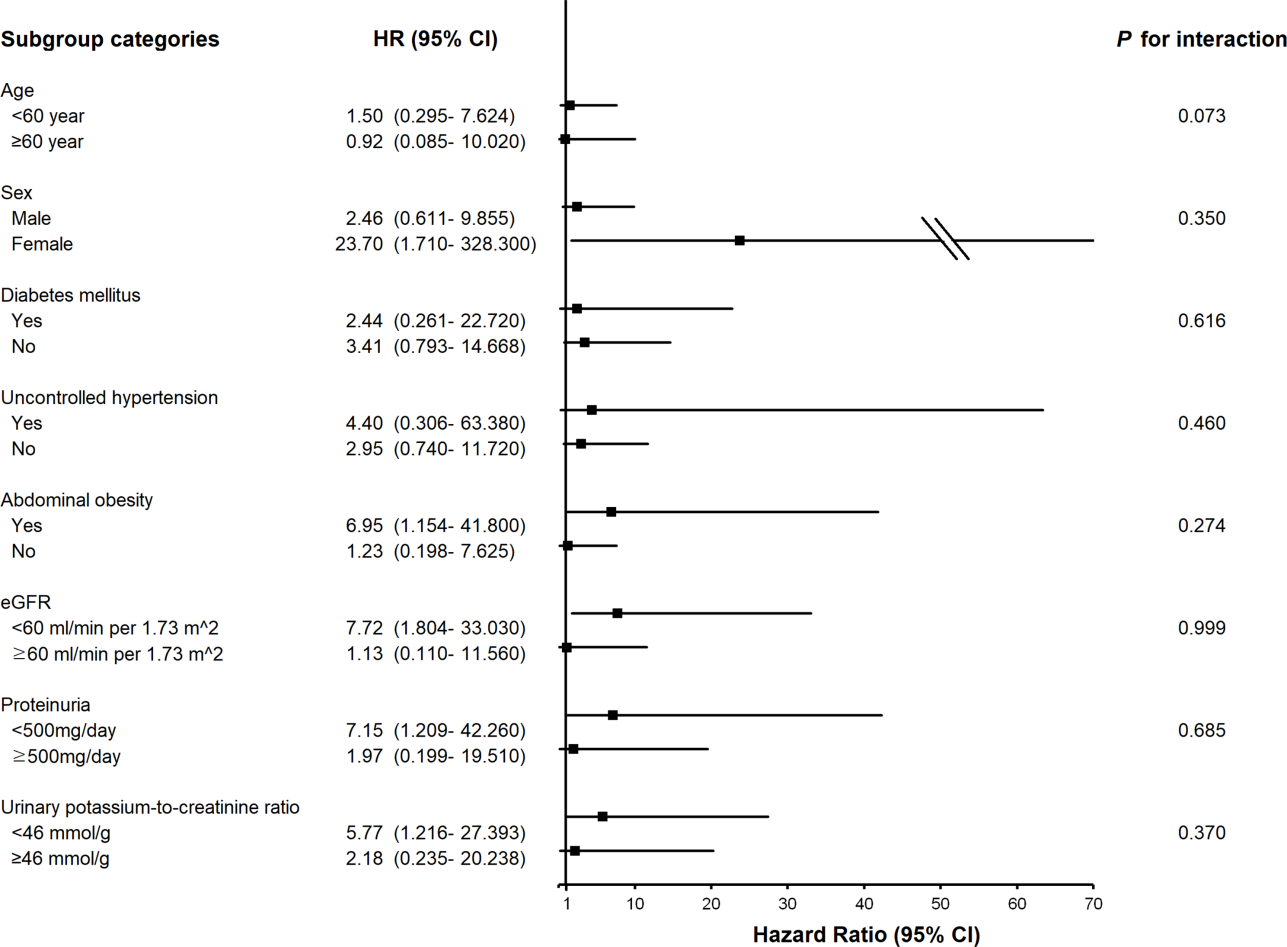
Subgroup association of measured 24-h urinary sodium excretion with the composite outcome of cardiovascular event and all-cause death. HR, hazard ratio; eGFR, estimated glomerular filtration rate.

To identify modification effects of subgroups on the relationship between urinary Na excretion and the composite outcome, subgroup analyses were conducted according to age (< 60 or ≥ 60 years), sex (male or female), presence of DM (yes or no), uncontrolled hypertension (yes or no), abdominal obesity (yes or no), eGFR (< 60 or ≥ 60 ml/min per 1.73 $m^{2}$ ), proteinuria (< 500 or ≥ 500mg/day), and urinary potassium-to-creatinine ratio (< 46 or ≥ 46 mmol/g). As a result, p values for interactions were > 0.05 for all subgroups, suggesting that the increased risk of the composite outcome associated with high urinary Na excretion was consistent regardless of these factors. The association between urinary Na excretion and the composite outcome was more significant in subgroups of patients with abdominal obesity, female, lower eGFR (< 60 ml/min per 1.73m^2^), no overt proteinuria, or a lower urinary potassium-to-creatinine ratio (< 46 mmol/g) (**Figure 2**).

### Sensitivity analyses

To perform sensitivity analyses, we used the estimated 24h urinary Na excretion using PAHO formula with random urine Na and creatinine. A spline curve was applied to identify HRs for the composite outcome and MACE, adjusting for age, sex, and BMI. As a result, there was a positive association between estimated 24h urinary Na excretion and the composite outcome in the spline curve (**Supplementary Figure 2A**). Also, in terms of MACE, the spline curve showed a positive relationship between estimated 24h urinary Na excretion and MACE (**Supplementary Figure 2B**).

## Discussion

In our study, measured urinary Na excretion ≥ 8.0g/day was associated with a significantly increased risk of the composite outcome, defined as the occurrence of CV events or all-cause death, and increased risk of MACE. The risk of the composite outcome from high salt intake was pronounced in subgroups of patients with abdominal obesity, female gender, lower eGFR (< 60 ml/min per 1.73m^2^), no overt proteinuria, or a lower urinary potassium-to-creatinine ratio (< 46 mmol/g). Our results were solid because we showed similar results for estimated 24h urinary Na excretion in a sensitivity analysis.

In prior studies analyzing the effect of dietary Na intake on CV outcomes, the study groups either mirrored the general population (13, 14), consisted of subjects with specific conditions such as prehypertension (22) or overweight (23), or included only patients with certain stages of CKD such as CKD stage III-IV (24, 25). Other studies with CKD patients (24–26) were limited by their small number of subjects (n< 500) and might be insufficient to represent the various etiologies of CKD. Similar to the CRIC study that analyzed 3,757 patients (12), our study included a large number of CKD patients. In addition, our analysis included patients with various etiologies of CKD and all stages of CKD and represented the entire population of CKD patients. However, compared to the CRIC study, our study participants were younger, with an average age of 55 years, while the average age of the subjects in the CRIC study was 58.2 years. Also, the present study cohort exhibited a lower number of composite CV outcomes, with 205 events (10.5%) among 1,937 participants, whereas there were 804 events (21.4%) among the 3,757 participants in the CRIC study. However, the mean 24h Na excretion was similar, with 3,657 mg/day in our study and 3,701 mg/day in the CRIC study.

In other studies, the cutoff points of 24h urinary Na excretion above which the hazard ratios for composite CV outcomes increase in CKD patients were 4 g, 4.5 g, and 4.8 g per day (12, 15, 16). In our study, the cutoff point was higher than in other studies, at 8.0 g/day. The reason for this difference among studies is thought to be the variation in study subjects (27–29). The risks of CKD progression and CV diseases vary across countries, even after adjusting for age, comorbidities, and laboratory markers (17). Therefore, variation among study participants will contribute to differences in the level of urinary Na excretion that significantly affects the risk of CV outcomes.

In our study, the reference group was that with the lowest Na excretion (24h urine Na excretion< 2.0g/day). For composite CV outcomes, compared to the reference group, hazard ratios showed a slight and not significant increase for the second, third, and fourth groups (24h urine sodium excretion< 8.0g/day) with values of 1.47, 1.27, and 1.19, respectively, in model 3. However, the hazard ratio for the fifth group (24h urine Na excretion ≥ 8.0g/day) showed a sharp and significant increase to a value of 3.29. Our study consequently showed a positive relationship between urinary Na excretion and CV outcomes. In one study, the lowest Na group (urinary Na excretion< 2.3g/day) showed the lowest hazard ratio, showing positive associations similar to our study (22). However, in the CRIC study, the lowest Na group (urinary Na excretion< 2.894g/day) exhibited a higher hazard ratio than the reference group, showing a J-shaped association between urinary Na excretion and composite CV outcomes (12). Many studies have shown such a positive (22, 23) or J-shaped association (12, 14), continuing the debate. Further studies should be performed to identify the risks in groups with very low urinary Na excretion.

In the group with the highest urinary Na excretion (24h urinary Na excretion ≥ 8.0g/day), the risk of composite CV outcomes was 3.29 times higher than that of the reference group (24h urinary Na excretion< 2.0g/day). One of the mechanisms for this result is the positive association between urinary Na excretion and blood pressure (30), and high blood pressure can contribute to CV diseases (31). CKD patients are more salt-sensitive than the general population (32), and increased blood pressure was pronounced in subjects with hypertension and a high-salt diet (33). Most of the participants in our study had hypertension, so participants in the group with the highest urinary Na excretion were more susceptible to increases in blood pressure. Also, there is a positive association between urinary Na excretion and left ventricular mass, and hypertension can reinforce this association (34). Furthermore, urinary Na excretion is associated with fluid retention, left ventricle wall thickness, and oxidative stress (35–37), all of which are can contribute to the occurrence of CV diseases (38–40).

The increased risk of CV events in patients with a high-salt diet was pronounced in female patients. Women are more likely than men to experience increase in left ventricle wall thickness (41) and age-related concentric remodeling (42), which are risk factors for CV disease (39). Also, in terms of hormones, estrogen suppresses smooth muscle cell proliferation (43) and attenuates cardiomyocyte hypertrophy (44), acting as a cardioprotective factor (45). Therefore, menopause is considered a risk factor for CV disease (43), and about 63% of the female participants in this study were older than 50 years, which is the average age of menopause in Korean women (46). Furthermore, there are risk factors of CV disease related to the female reproductive cycle, which are unique to women (47). These would lead to more significance between a high-salt diet and CV outcomes in women. Also, the risk of CV events was pronounced in patients with abdominal obesity. Obesity is related to metabolic syndrome (48) and inflammation (49), which can contribute to increased risk of CV disease.

Among the baseline characteristics, the association between SBP and urinary Na excretion is interesting. In the section where urinary Na excretion<8g/day, the higher the urinary Na excretion, the higher the SBP. However, in the section where urinary Na excretion ≥8g/day, SBP is lower than in the urinary Na excretion between 6 and 8 g/day. This is thought to be because there are many young people and many people with good renal function in the section where urinary Na excretion ≥8g/day.

Among all patients, 612 (31.6%) patients were using diuretics. The median urinary Na excretion value of patients using diuretics was 153.9 mEq/day, higher than the median value of 150.5 mEq/day for patients not using diuretics (**Supplementary Table 1**). Loop diuretics inhibit Na reabsorption by acting on the thick ascending limb of the loop of Henle (50), thiazide diuretics on the distal convoluted tubule (51), and potassium-sparing diuretics on the aldosterone-sensitive distal nephron (52). These induce natriuresis, resulting in urinary Na excretion. Among the patients with diuretics, the highest urinary Na excretion values were in the order of the group using thiazide, the group using potassium-sparing diuretics, and the group using loop diuretics.

The strength of our study is the use of a long-term cohort with a large and ethnically homogenous population. In the KNOW-CKD study, a large number of subjects was evaluated for CV events and CKD progression during long-term follow-up. Also, measuring 24h urinary Na excretion is known to be a more accurate method for assessing dietary Na intake (53). While some studies used random urine or the dietary recall methods to estimate dietary Na intake (13, 14, 23), we used measurement of 24h urine Na excretion to estimate dietary Na intake.

Our study had several limitations. First, our study was observational, so a cause-and-effect relationship could not be accurately investigated. Second, we used a single measurement of 24h urinary Na excretion, instead of repeated 24hour urinary Na excretion measurements (12, 22). Nevertheless, we also conducted a sensitivity analysis with estimated 24h urinary Na excretion from random urine samples in order to supplement the limitation. The study results were derived from a small number of events in the smallest number of groups. Therefore, the statistical power of the results was weak. However, since the cohort is a prospective cohort, more reliable results can be obtained as more data are accumulated.

In conclusion, a high-salt diet is associated with elevated risk of CV outcomes in CKD patients. Our study suggests that urinary Na excretion can be a predictor of the occurrence of CV diseases.

## Data availability statement

The original contributions presented in the study are included in the article/**Supplementary Material**. Further inquiries can be directed to the corresponding author.

## Ethics statement

The studies involving humans were approved by The Institutional Review Boards of Seoul National University Hospital (1104-089-359), Seoul National University Bundang Hospital (B-1106/129-008), Yonsei University Severance Hospital (4-2011-0163), Kangbuk Samsung Medical Center (2011-01-076), Seoul St. Mary’s Hospital (KC11OIMI0441), Gil Hospital (GIRBA2553), Eulji General Hospital (201105-01), Chonnam National University Hospital (CNUH-2011-092), and Busan Paik Hospital (11-091). The studies were conducted in accordance with the local legislation and institutional requirements. The participants provided their written informed consent to participate in this study.

## Author contributions

The specific contribution of each author is as follows. SCK, MK, and K-HO designed the study. SCK and MK analyzed and interpreted the results and drafted the manuscript. HR, SK, JHK, and EK analyzed and interpreted the data. YJ and JK advised and assisted in the statistical analysis. Y-SK, SWK, and YHK reviewed and edited the manuscript. K-HO conceived the study, analyzed the results, interpreted the data and reviewed the manuscript. All authors contributed to the article and approved the submitted version.
