# Supplementary material for: Measured sodium excretion is associated with cardiovascular outcomes in non-dialysis CKD patients: results from the KNOW-CKD study
Source: Front Nephrol. 2023 Aug 25;3:1236177. doi: 10.3389/fneph.2023.1236177 (PMC10479682; doi:10.3389/fneph.2023.1236177)
Supplement: Supplementary file 1 [file DataSheet_1.pdf]

Measured sodium excretion is associated with cardiovascular outcomes in non-dialysis CKD patients: Results from the KNOW-CKD study

Corresponding author:

Kook-Hwan Oh (khoh@snu.ac.kr)

Contents

Supplemental Figure 1. ·· 2

Supplemental Figure 2. ·· 3

Supplemental Table 1. ·· 4

Supplemental Figure 1. Flow diagram of study cohort.

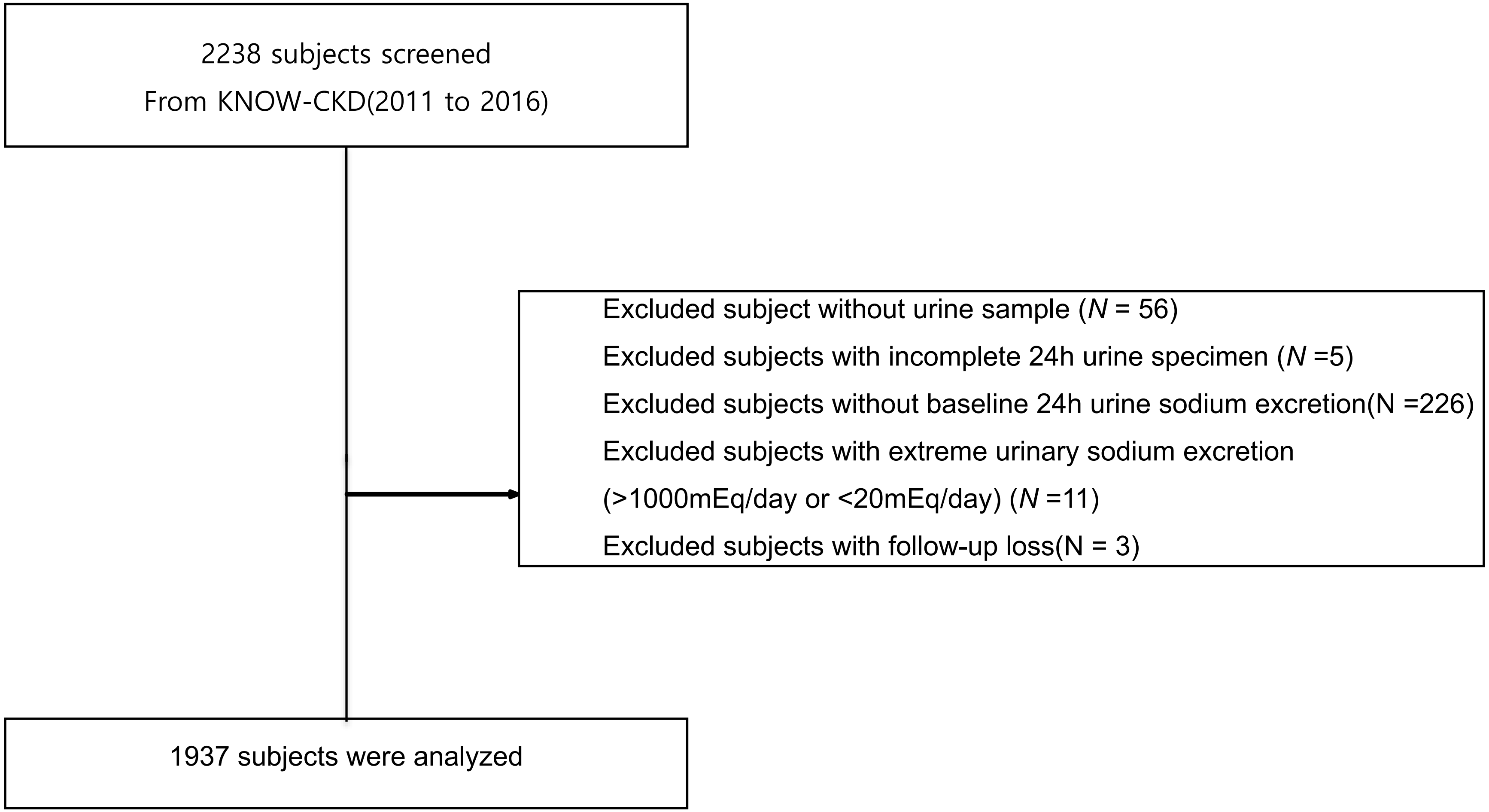

Supplemental Figure 2. Association of estimated 24-h urinary sodium excretion with HR for the composite outcome of cardiovascular event and all-cause death (A) and major adverse cardiac events (B), after adjustment for age, sex and BMI.

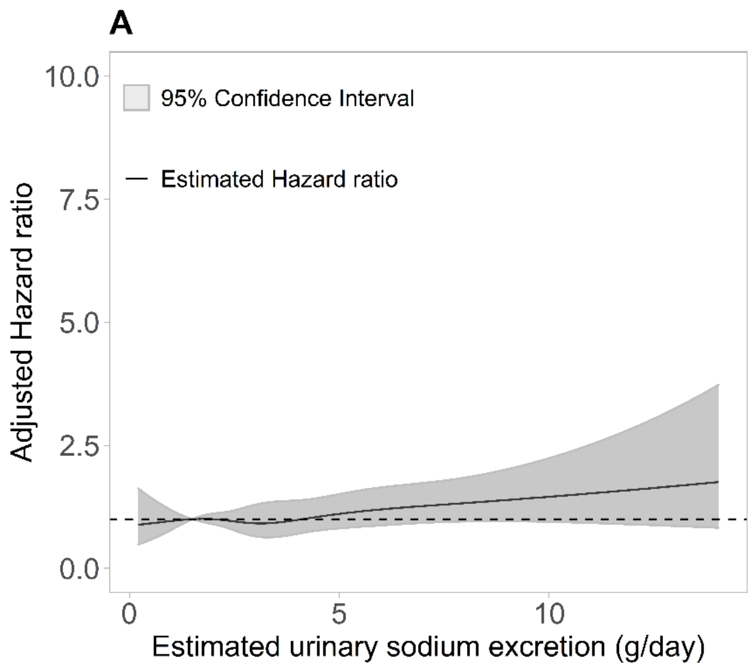

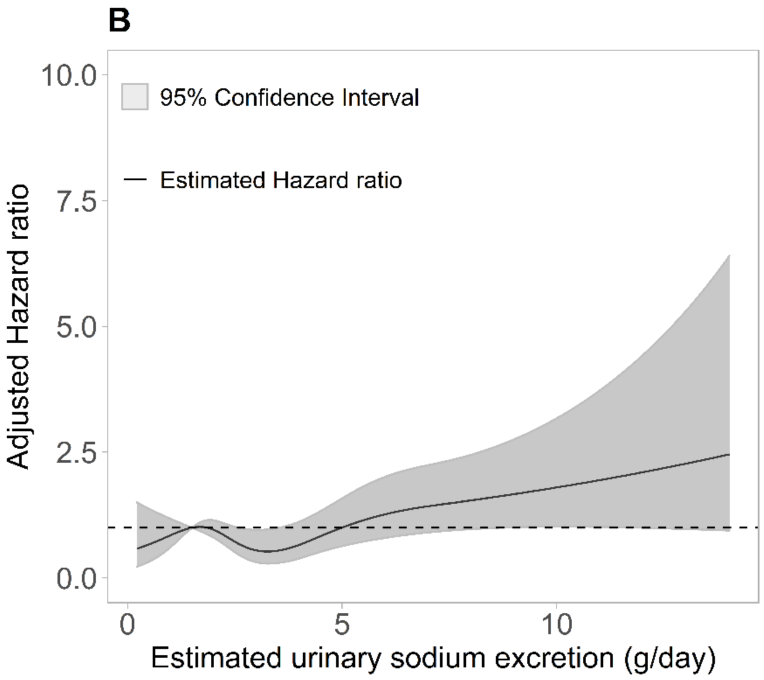

Supplemental Table 1. Urinary sodium excretion according to the type of diuretics.

Variable	Diuretics (n = 612)	Loop diuretics (n = 346)	thiazide (n = 224)	Potassium-sparing diuretics (n = 42)
24h measured urinary Na excretion, mEq/day	153.9 [113.0;202.1]	145.4 [104.1;190.8]	170.1 [119.2;223.9]	156.9 [117.0;190.6]
